# Origin of the Autophagosomal Membrane in Plants

**DOI:** 10.3389/fpls.2016.01655

**Published:** 2016-11-04

**Authors:** Xiaohong Zhuang, Kin Pan Chung, Liwen Jiang

**Affiliations:** ^1^School of Life Sciences, Centre for Cell and Developmental Biology and State Key Laboratory of Agrobiotechnology, The Chinese University of Hong KongShatin, Hong Kong; ^2^The Chinese University of Hong Kong Shenzhen Research InstituteShenzhen, China

**Keywords:** autophagy, autophagosome, membrane origin, ER, membrane contact site

## Abstract

During autophagy, cargo molecules destined for degradation are sequestrated into a double-membrane structure called autophagosome, which subsequently fuses with the vacuole. An isolation membrane structure (also called the phagophore) initiates from the platform termed PAS (phagophore assembly site or preautophagosomal structure), which then elongates and expands to become the completed autophagosome. The origin of the membrane for autophagosome formation has been extensively investigated but remains an enigma in the field of autophagy. In yeast and mammalian cells multiple membrane sources have been suggested to contribute to autophagosome formation at different steps, from initiation through expansion and maturation. Recent studies in plants have provided a significant advance in our understanding of the conserved role of autophagy and the underlying mechanism for autophagosome formation. Here, we will discuss and evaluate these new findings on autophagosome formation in plants, with a particular focus on the origin of plant autophagosomal membranes.

## Introduction

Macroautophagy (hereafter simply autophagy) is a conserved degradative pathway for the removal of cytoplasmic materials in eukaryotic cells, and is characterized by the formation of a double-membrane structure called the autophagosome (Mizushima and Komatsu, 2011). During the past decades, our understanding of the physiological role of autophagy in plants has been greatly extended, and now includes information on its primary function under stress or starvation conditions for bulk degradation of cytoplasmic cargo (non-selective autophagy), and on its emerging role in the specific degradation of defined macromolecules or organelles (selective autophagy; Liu and Bassham, 2012). These studies support a conserved and essential role for autophagy in the life of plants.

Autophagosome formation is orchestrated by a subset of autophagy-related (ATG) proteins, which are coordinated in a spatio-temporal manner with most of the components being dissociated and recycled back from the completed autophagosome (Lamb et al., 2013). In yeast and mammals, diverse membrane sources have been proposed to contribute to autophagosome formation, including the endoplasmic reticulum (ER), mitochondria, ER-mitochondria contact sites, the ER-Golgi intermediate compartment (ERGIC), Golgi apparatus, ATG9 vesicles, recycling endosomes, and the plasma membrane (PM; Axe et al., 2008; Hayashi-Nishino et al., 2009; Matsunaga et al., 2010; Ravikumar et al., 2010; Yamamoto et al., 2012; Ge et al., 2013; Hamasaki et al., 2013; Puri et al., 2013).

In plants, a number of core autophagy-related (ATG) counterparts have been implicated in selective and/or non-selective autophagy (Liu and Bassham, 2012). Conserved autophagosome-related structures, including the phagophore and the completed double-membrane autophagosome, have been characterized at the ultrastructural level in plants (Isono et al., 2010; Zhuang et al., 2013; Le Bars et al., 2014; Gao et al., 2015; Lin et al., 2015; Spitzer et al., 2015). However, the underlying mechanism(s) of autophagosome biogenesis in plants remain largely unexplored. Here we focus on selected recent studies into autophagosome biogenesis in plants especially in regard to the origin of its membrane.

## Autophagosome Initiation: Er Involvement?

The origin of autophagosome is divergent as derived from studies in different organisms. In yeast, it has been reported that ATG9 vesicles are essential for the nucleation of the phagophore, while in mammalian cells, an “omegasome” structure that arises from an ER subdomain appears to be responsible for the phagophore initiation (Mizushima et al., 2011; Lamb et al., 2013). The formation of the omegasome requires the recruitment of phosphoinositide 3-kinase (PI3K) complex for the production of phosphatidylinositol 3-phosphate (PI3P) which then recruits its downstream effectors to facilitate the membrane remodeling process (Matsunaga et al., 2010). In contrast, information about PAS in plants is limited. For example, whether the plant autophagosome utilizes a *de novo* assembly model as described in yeast, or the maturation model that suggested the autophagosome is derived from a pre-existing membrane in animals, remains unclear. Additionally, some of the ATG counterparts for phagophore initiation have not been identified or characterized, in particular those distributed on the initiation site of the phagophore such as ATG14 and ATG16.

**FIGURE 1:**
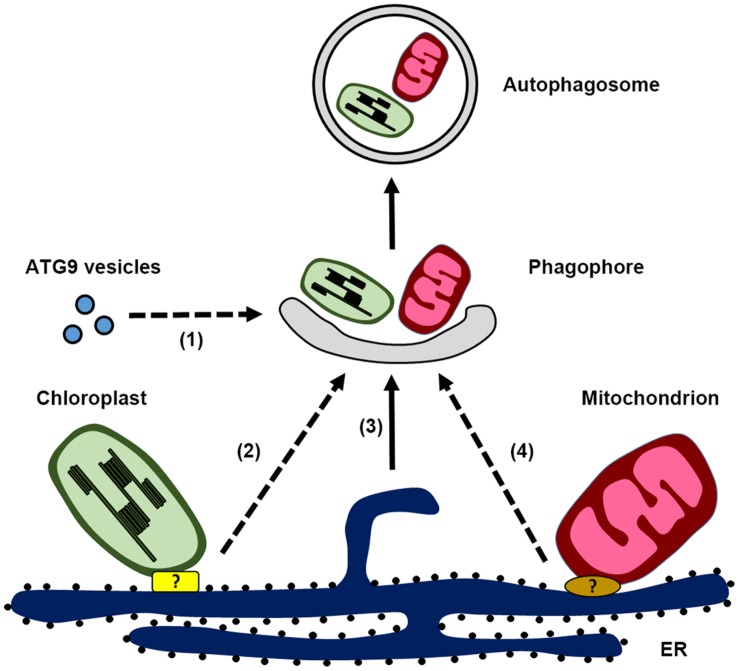
**Schematic illustration of autophagosome biogenesis in plant cells, highlighting the possible membrane sources for phagophore formation: (1) ATG9 vesicles, (2) endoplasmic reticulum (ER)-chloroplast contact site, (3) ER, and (4) ER-mitochondria contact site.** Potential protein complex responsible for the ER-chloroplast contact site and ER-mitochondria contact site are labeled with the question mark.

Despite the limited information in plant autophagosome biogenesis, recent findings provide new evidence that plant autophagosomes might originate from the ER (**Figure 1**). Observations made under ER stress show that the autophagosomal membrane is associated with the ER (Liu et al., 2012; Yang et al., 2016), although this kind of association could reflect the possibility that the ER is being engulfed in autophagosome for subsequent degradation. Further evidence for the involvement of the ER in autophagosome biogenesis was provided by tracing the dynamics of ATG5-GFP upon autophagic induction (Le Bars et al., 2014). In this study, it is nicely shown that the ATG5-labeled toroidal domain develops into crescent-like expanding phagophore at the outer surface of the ER, although a direct connection exists between phagophore and the ER is still uncertain. Moreover, a close association between the ER membrane and another autophagosome-related protein, SH3P2, has been observed during autophagosome formation (Zhuang et al., 2013; Zhuang and Jiang, 2014). Electron microscopy analysis shows that SH3P2-positive phagophores are often accompanied with ER fragments on both sides. This is quite reminiscent of the PI3P-enriched omegasome structures described in animals, from which cup-shaped ER cisternae are formed and invaginated to produce the isolation membrane (Axe et al., 2008). Consistent with this is the observation that the PI3P inhibitor wortmannin abolish the formation of either ATG5-GFP or SH3P2-GFP labeled punctae, suggesting a conserved role for PI3K complex function during autophagy. Although the molecular mechanisms of most ATG proteins have not been well investigated in plants, characterization of the subcellular localization of ATG proteins during autophagy should provide significant insights into the process of phagophore formation, as well as demonstrating membrane continuity between the phagophore and the ER. In addition, COPI and COPII machineries for trafficking between ER and Golgi have been implicated to be involved in autophagosome formation (Razi et al., 2009). It is possible that autophagosome formation may require these ER-related machineries as well, like the recently identified plant-unique COPII machinery (Zeng et al., 2015).

## Autophagosome Expansion and Maturation: Cross Talk with Endosomes?

In mammalian cells, autophagosome undergoes a further maturation step by fusing with endosomes to form an amphisome (Lamb et al., 2013). In plants, such a fusion event between autophagosome and endosome has not been reported, but a crosstalk between endosomes and autophagosomes has been suggested (Zhuang et al., 2015). In several ESCRT-related protein mutants, accumulation of autophagosomes has been observed (Isono et al., 2010; Gao et al., 2015; Spitzer et al., 2015). Recently, it was reported that FREE1 or FYVE1, a unique ESCRT component that regulates MVB morphology (Gao et al., 2014; Kolb et al., 2015), interacts with the autophagosome-related protein SH3P2 to play a dual role in autophagosome formation (Gao et al., 2015). In wild-type *Arabidopsis* plants, the fusion event between the MVB and autophagosome is rarely detected. While in *free1* mutant there is a dramatic increase in the overlapping signals between autophagosome marker and MVB marker. In addition, the delivery of autophagosomes into the vacuole is blocked in *free1* mutant. It is therefore suggested that this crosstalk between the ESCRT component FREE1 and SH3P2 may promote the fusion between the autophagosome and endosome for autophagosome expansion or maturation.

Additionally, other studies indicated recycling endosomes may contribute to autophagosome maturation. Retromer, which is known to regulate receptor recycling from endosomes to the trans-Golgi network (TGN) in animals, has recently been reported to be involved in autophagy (Orsi et al., 2012; Popovic and Dikic, 2014; Zavodszky et al., 2014). It is claimed that autophagy defects in the retromer mutant might be caused by the missorting of ATG9 vesicles between the PAS and non-PAS pool, which is dependent on the functional retromer. Although the location and function of retromer in plants remains controversial (Oliviusson et al., 2006), recent study showed that the vacuolar delivery of autophagosome is impaired in the absence of a retromer subunit VPS35 (Munch et al., 2015). In addition, another study in the pathogen *Magnaporthe oryzae* provides a novel insight into the role of the retromer complex in recycling of lipidated MoAtg8 during autophagosome formation (Zheng et al., 2015). In this study, deletion of one retromer subunit, MoVPS35, leads to the mislocalization of RFP-MoAtg8 into the vacuole and failure in recycling from the autolysosome. Intriguingly, MoVps35 interacts with MoAtg8 and localizes to the periphery of vacuoles/autolysosomes with other retromer subunits including MoVps26 and MoVps29.

Other fusion regulators that are involved in autophagy for either endosome or vacuole have also been reported, including Rab-GTPase (RABG3f) and SNARE proteins (VTI family; Surpin et al., 2003; Kwon et al., 2013). Future investigations may reveal how these conventional endosomal regulators cooperate with the autophagic machinery during autophagosome formation, as well as whether endosomes would contribute as the autophagosomal membrane source in plants.

## Atg9 Vesicles

ATG9 vesicles are another potential membrane source for autophagosome formation that have been extensively characterized in yeast and mammalian cells (Lamb et al., 2013). As the sole membrane-spanning protein, it is suggested that ATG9 may play a role in delivering membrane/lipid onto the nascent phagophore, as ATG9 deficient mutants in yeast or mammal fail to form autophagosomes (Mari et al., 2010; Orsi et al., 2012; Yamamoto et al., 2012). In both yeast and mammalian cells, ATG9 is often found on Golgi-derived vesicles in the cytoplasm. Upon autophagic induction, ATG9 vesicles accumulate at the PAS in an ATG1-dependent manner (Mari et al., 2010; Yamamoto et al., 2012). In animals, ATG9 vesicles transit from the Golgi to PAS during autophagy, which recruit ATG8 and the PI3P effector, ATG18 (WIPI in animals). In addition, mammalian ATG9 was found to traffic via the PM onto recycling endosomes and colocalize with ATG16L1 (Puri et al., 2013). In addition, a number of regulators that are involved in the trafficking of ATG9 have been identified, and disruption of ATG9 trafficking between PAS and non-PAS pool interferes with autophagosome formation (Lamb et al., 2013). Recently, evidence showing that ATG9 vesicles together with ER tubules make up a tubulo-vesicular platform for the origin of the autophagosome, places ATG9 at an early event in the nucleation of the phagophore at the ER membrane (Karanasios et al., 2016).

In plants, a homolog of ATG9 has been identified and *atg9* mutants also display an early leaf senescence phenotype that is similar to other *atg* mutants (Hanaoka et al., 2002; Guiboileau et al., 2012). In the *atg9* mutant, less autophagic bodies were detected when cells were treated with inhibitors to block vacuolar degradation (Inoue et al., 2006; Shin et al., 2014). It therefore seems that ATG9 is not required for the entire autophagic flux during nitrogen starvation, as knockouts of ATG9 only partially suppress the turnover of YFP-ATG8a (Shin et al., 2014). However, the identity of ATG9 vesicles in plant has not been clarified as yet. It would be interesting to know if ATG9 vesicles would play a role in nucleating phagophore and/or contribute membranes to the growing autophagosome in plants (**Figure 1**). Moreover, how ATG9 coordinates with other molecules to function in autophagosome formation remains unexplored in plants. These are the essential questions to be addressed in the future to advance our understanding of the role of ATG9 in plant autophagosome biogenesis.

## Autophagosome Membrane Origin in Selective Autophagy: Membrane Contact Sites?

A role for selective autophagy in plants has been established in recent years, especially in the degradation of the ER, mitochondria, chloroplasts, peroxisomes, and exocyst-positive organelle as well as TSPO-binding proteins for cellular homeostasis (Wang et al., 2010; Floyd et al., 2012; Li and Vierstra, 2012; Michaeli and Galili, 2014; Veljanovski and Batoko, 2014; Lin et al., 2015; Xie et al., 2015). However, the mechanism of autophagosome initiation for selective autophagy has not been well characterized. Recent studies in yeast and animals revealed the involvement of ER-mitochondria membrane contact sites (MCS) in mediating selective/non-selective autophagy (Hamasaki et al., 2013; Bockler and Westermann, 2014). In regarding to the essential role of the MCSs in lipid delivery and membrane tethering, the MCSs may serve as an ideal platform for the autophagosome initiation during selective autophagy (Phillips and Voeltz, 2016). Extensive contacts that ER makes with other organelles in plants has also been observed, as well as plant-specialized MCS structure (Hawes et al., 2015; Perez-Sancho et al., 2016). For example, chloroplast is dynamically connected with the ER via extending stromules (Mitsuhashi et al., 2000; Schattat et al., 2011). Recently, plant specific regulators for plasmodesmata, the ER-PM contact site, have been reported (Wang et al., 2016). In the following parts, we will use mitophagy and chlorophagy as examples to discuss the potential role of the MCS for selective autophagy in plants.

## Mitophagy: Er-Mitochondria Contact Site?

In yeast, ER-mitochondria contact is mediated by the ER-mitochondria encounter structure (ERMES), which is composed of Mmm1, Mdm10, Mdm12, and Mdm34 (Kornmann et al., 2009). Recent study suggested that ERMES is an important factor contributing to selective degradation of mitochondria through mitophagy (Bockler and Westermann, 2014). Upon autophagic induction, ERMES colocalizes with autophagic machinery proteins such as ATG5 and ATG8. Intriguingly, mutants lacking functional ERMES subunits show strong defects in mitophagy but not bulk autophagy, indicating a specific role for ER-mitochondria contacts in mitophagosome formation. ERMES have also been suggested to have a role in lipid transfer between membranes (Voss et al., 2012). It is speculated that ERMES may promote lipid delivery from the ER to the growing phagophore surrounding the mitochondria to provide sufficient membrane materials.

Recent studies in mammalian cells further demonstrate the importance of ER-mitochondria contact in mitophagy. Impaired mitochondria are found to be associated with the ER, while LC3 recruitment onto the ER-mitochondria contact regions is also observed (Yang and Yang, 2013; Wu et al., 2016). Previously, it is reported that ATG14, a key component for phagophore initiation, mobilizes to the mitochondria-associated ER membrane (MAMs) fraction together with the omegasome marker DFCP1 and the ER-resident SNARE protein syntaxin 17 (STX17; Hamasaki et al., 2013). Moreover, inhibition of the translocation of ATG14 and DFCP1 on the MAM compartment by interfering with the ER-mitochondria contact site prevents proper autophagosome formation. These findings support that the ER-mitochondria contact site serves as an essential platform for autophagosome formation, particularly during mitophagy.

Recent evidence indicates that a conserved association between ER and mitochondria occurs in plant as well (Jaipargas et al., 2015; Mueller and Reski, 2015). Based on live-cell imaging data in *Arabidopsis*, it is suggested that membrane continuity between ER and mitochondria exist, as the mitochondrial morphology is dependent upon the fusion and fission events which are correlated with ER dynamics (Jaipargas et al., 2015). In regard to the cooperative role of ER and mitochondria in biosynthetic pathways and the exchange of phospholipids, it is possible that plant ER-mitochondria contact site might play a role during mitophagy for phagophore formation as observed in yeast and animals (**Figure 1**). Interestingly, a recent study showed that *Arabidopsis* ATG11 colocalizes with the mito-tracker and plays a direct role in mitophagy by interacting with ATG8 as a cargo receptor (Li et al., 2014). In yeast, ATG11 interacts with the membrane protein ATG32 and the mitochondrial fission machinery during mitophagy (Mao et al., 2013). Since there is no homologs of yeast ERMES identified in plants yet, identification of the molecular machinery that regulates the ER-mitochondria connectivity as well as their correlation with the ATG machinery would definitely provide valuable information for plant mitophagy.

## Chlorophagy: Er-Chloroplast Contact Site?

The degradation of damaged chloroplasts and the subsequent recycling of nutrients is important for plants to cope with stress and different developmental stages, especially during leaf senescence (Wada et al., 2009; Ishida et al., 2014). Leaf starch degradation during the night is also reported to be mediated by the autophagic machinery, and silencing of autophagy-related genes such as ATG6 results in excess starch accumulation (Wang et al., 2013). Up till now, multiple degradation pathways have been proposed for the turnover of chloroplast proteins. These include the senescence-associated vacuole (SAV) pathway (Otegui et al., 2005; Martinez et al., 2008), the chloroplast vesiculation (CV) containing vesicle pathway (Wang and Blumwald, 2014), the Rubisco-containing body (RCB) pathway (Chiba et al., 2003; Ishida et al., 2008; Izumi et al., 2010), the ATI-plastid (ATI-PS) body pathway (Michaeli et al., 2014), and the whole-chloroplast autophagy pathway (Wittenbach et al., 1982; Minamikawa et al., 2001). In the following section, we will focus on discussing the autophagy-dependent RCB pathway.

The RCB is a double-membrane bound structure derived from the chloroplast, which contains chloroplast stromal proteins but not thylakoids. RCBs are commonly found in senescent leaves in which they will be delivered to the vacuole for degradation and thus piecemeal degradation of chloroplasts via RCB is achieved (Chiba et al., 2003; Ishida et al., 2008; Wada et al., 2009; Izumi et al., 2010). Molecular mechanism for the formation of RCB is still not available, but the degradation of stromal and photosynthetic proteins has been suggested to be dependent on the autophagic machinery, which is evidenced by compromised degradation of these proteins in *atg4a4b-1*, *atg5*, and *atg7* mutants (Wada et al., 2009; Lee et al., 2013; Sakuraba et al., 2014). On the other hand, arrested phagophores and RCB-like vesicles accumulated in the cytoplasm in the ESCRT machinery subunit *chmp1* mutant (Spitzer et al., 2015). In addition, autophagosomal marker ATG8 was reported to be associated with chloroplast protrusion and stromules (Ishida et al., 2008; Spitzer et al., 2015). Interestingly, chloroplast protrusions and stromules are found to be more abundant in *atg5* mesophyll than in wild-type plants (Ishida et al., 2008), indicating sequestration of protruding stromules via the isolation membrane may contribute to RCB formation.

Several studies have indicated a dynamic association between the chloroplast and ER, which is evidenced by the observation of the branched ER tubules at chloroplast surface or extending stromule branching coincides with the ER tubules (Mitsuhashi et al., 2000; Schattat et al., 2011). It raises the possibility that the ER-chloroplast contact site may function as a platform for RCB biogenesis (**Figure 1**). Therefore, disruption of the autophagic machinery leads to protruding stromules, which are unable to form RCB, while suppression of autophagosome maturation in the *chmp1* mutant results in accumulation of phagophores associated with the chloroplast. ER-chloroplast contact sites have been proposed to be essential for lipid trafficking, which is supported by the observation that several lipid processing enzymes such as PC synthase, TGD2 and TGD4 are detected at ER-chloroplast membrane junctions (Wang et al., 2012). It would be interesting to test whether disruption of ER-chloroplast lipid trafficking will affect the RCB formation.

## Challenge and Future Perspective in Plant Autophagosome Biogenesis

Although studies on plant autophagy have only got started, tremendous progress has been made on different aspects from its physiological role to the identification of unique plant autophagy machineries and their regulators. Multiple lines of evidence have suggested that the plant autophagosome is probably developed from the ER or ER-related membranes. The MCS between ER and other organelles such as mitochondria and chloroplast may provide platform for autophagosome biogenesis. However, there are still many questions waiting to be solved on plant autophagosome biogenesis: What is the nature of the phagophore and ATG9 vesicles in plants? Do different membrane sources contribute to autophagosome formation in a condition-dependent manner? Future efforts in elucidating the molecular mechanism among the autophagy networks and in-depth investigations into autophagosome-related structures should provide important insights into our understanding of plant autophagosome biogenesis.

## Author Contributions

XZ, KPC, and LJ designed the concept and the organization of the manuscript; XZ and KPC wrote the manuscript; LJ edited the manuscript.

## Conflict of Interest Statement

The authors declare that the research was conducted in the absence of any commercial or financial relationships that could be construed as a potential conflict of interest.
